# Cardiovascular Consequences of Late-Gestational Hypoxia in Adult Female Rat Offspring and the Effects of Maternal Ergothioneine Treatment

**DOI:** 10.33549/physiolres.935760

**Published:** 2026-06-01

**Authors:** Katarina BABARIKOVA, Diana CVIKOVA, Hana MAUER SUTOVSKA, Pavel SVITOK, Romana KOPRDOVA, Michaela PIESOVA, Mojmir MACH, Michal ZEMAN, Lubos MOLCAN

**Affiliations:** 1 Department of Animal Physiology and Ethology, Faculty of Natural Sciences, Comenius University Bratislava, Bratislava, Slovak Republic; 2 GYN – FIV a.s., Centre for Gynaecology and Assisted Reproduction, Bratislava, Slovak Republic; 3 Institute of Experimental Pharmacology and Toxicology, Centre of Experimental Medicine of the Slovak Academy of Sciences, Bratislava, Slovak Republic

**Keywords:** Ergothioneine, Prenatal hypoxia, Female, Cardiovascular, Telemetry

## Abstract

Prenatal hypoxia (PH) predisposes offspring to cardiovascular diseases. While maternal antioxidant treatment can normalize blood pressure (BP) in offspring, it typically requires high doses that may harm both mother and fetus. The antioxidant ergothioneine (ERG), noted for its very low toxicity, has emerged as a promising candidate. Because PH-induced changes have been primarily studied in male offspring, this study was performed to investigate the impact of maternal ERG treatment and late-gestational PH on cardiovascular outcomes in adult female rat offspring. Pregnant Wistar rats were exposed to 10.5 % O_2_ for 4 h on gestational days (GDs) 19 and 20. Adult offspring were implanted with telemetry sensors for continuous BP and heart rate (HR) monitoring. In a second experiment, pregnant rats received ERG (35 mg/kg/day) from GD6 until delivery, and on GD20 were exposed to 10.5 % O_2_ for 12 h. Cardiac and renal protein expression in adult offspring was analysed by western blot. PH offspring exhibited lower HR and BP responses to restraint stress than did controls. PH increased the low-to-high frequency ratio (a marker of sympathovagal balance; P=0.051) and the standard deviation of normal-to-normal interbeat intervals (P=0.024). Despite normal cardiovascular parameters at rest, late-gestational PH programmed subtle yet lasting changes in sympathovagal balance and impaired cardiovascular reactivity to stress in adult female offspring. ERG upregulated β_1_-adrenergic receptor and endothelial nitric oxide synthase, particularly in the PH group. This selective effect highlights ERG’s potential as a targeted antioxidant intervention against harmful prenatal conditions.

## Introduction

Adverse conditions *in utero* are now recognized as important risk factors for cardiovascular disease in postnatal life. Prenatal hypoxia (PH) is a relatively common pregnancy complication, arising from various conditions including gestational diabetes mellitus [1], impaired placentation [2], or maternal hypoxemia [3]. In experimental models, chronic PH has been linked to increased sympathetic nerve activity [4], increased myocardial contractility [5], impaired circadian synchronization [6], macrophage infiltration in the subendothelium [7], endothelial dysfunction [8], glomerular hypertrophy and renal fibrosis [9]. Notably, even brief episodes of hypoxia during late gestation – when organogenesis is largely complete – have been shown to elevate blood pressure (BP) and sympathetic nerve activity in adult male rat offspring studied in adulthood [10,11].

The mechanisms underlying PH-induced alterations are complex, involving changes in the autonomic nervous system [10], vascular endothelium [12], and renin–angiotensin–aldosterone system [9]. Importantly, oxidative stress has been identified as a central mediator of these maladaptive changes. Maternal antioxidant treatment can protect offspring from adverse effects [12], but the high doses required may pose risks to both mother and fetus [13,14]. By contrast, the naturally occurring antioxidant ergothioneine (ERG) appears to be safe even at very high doses [15]. ERG has shown beneficial effects in models of complicated pregnancies, where it improved embryo quality [16] and prevented malformations associated with diabetic pregnancy [17]. However, its long-term impact on offspring remains to be established.

Although the relationship between PH and cardiovascular dysfunction is well established in male offspring, females remain substantially understudied. Emerging data suggest that females respond differently to prenatal insults, often exhibiting a delayed or attenuated pathology compared with males [18–20]. Crucially, the absence of hypertension or renal pathology in female offspring does not necessarily indicate a lack of programming effects. Instead, these effects may be buffered by protective mechanisms and only become apparent under additional contributing factors such as ageing [9] or dietary stress [21]. Understanding these sex-specific patterns is critical because females who appear unaffected may still be at increased risk of cardiovascular disease under certain conditions.

Given the scarcity of data on female offspring, the present study was performed to analyze the cardiovascular consequences of short-term PH in adult female rat offspring. We investigated whether PH females, similarly to males, develop hypertension associated with increased sympathetic nerve activity. Cardiovascular parameters – including heart rate (HR), BP, autonomic nervous system activity, and stress reactivity – were assessed *via* telemetry. Hearts and kidneys were collected to examine changes in protein expression. Furthermore, we investigated whether long-term maternal treatment with ERG could attenuate or prevent hypoxia-induced molecular alterations.

## Material and Methods

### Animals

The experiments were approved by the Ethical Committee for the Care and Use of Laboratory Animals and the State Veterinary and Food Administration of Slovak Republic (2680/11-221, 1043-3/2020-220), following the EU directive (2010/63/EU) and ARRIVE guidelines. Experimental procedures, including mating, breeding and sampling, were conducted at the Institute of Experimental Pharmacology and Toxicology (Centre of Experimental Medicine of the Slovak Academy of Sciences, Slovak Republic). Telemetry was performed at the Faculty of Natural Sciences (Comenius University Bratislava, Slovak Republic).

Wistar rats (Dobra Voda, Slovak Republic) were housed and mated as previously described [10]. GD0 was established by the presence of spermatozoa in the vaginal smear. Pregnant females were kept in transparent cages with food and water ad libitum under a regular 12-h light/dark cycle, with a controlled room temperature (21±2 °C) and humidity (55±10 %). After spontaneous delivery, the litter size was reduced to four males and four females. Female offspring were assigned to the experimental groups after weaning (21 days postpartum) using a single pup per litter rule.

### Experimental design

**Fig. 1:**
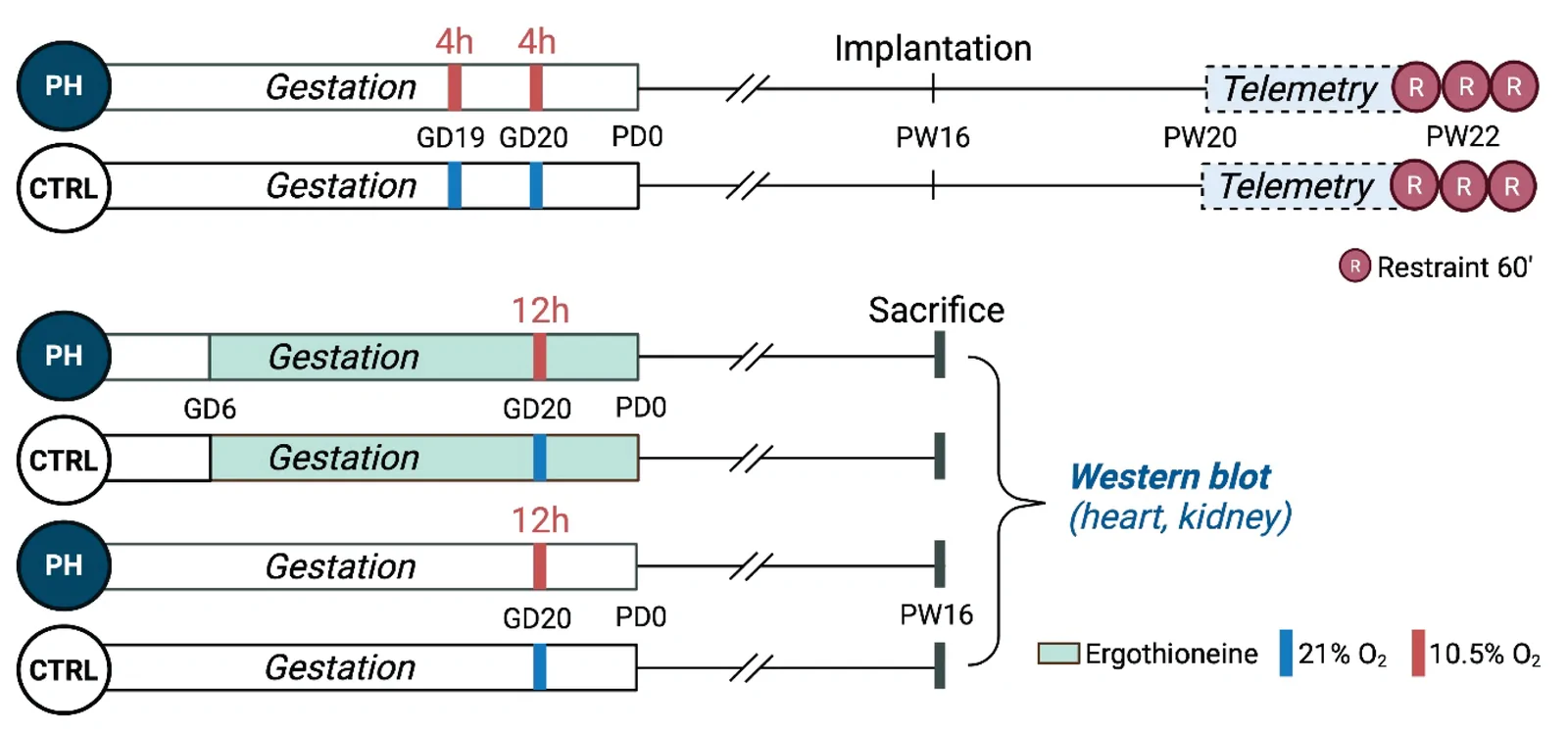
Schematic illustration of the experimental design. In Experiment 1, pregnant rats were exposed to normoxia or short-term hypoxia (10.5 % O_2_, 4 h/day on GD19-20), and cardiovascular function was assessed in adult female offspring using telemetry. In Experiment 2, pregnant rats received ERG (35 mg/kg/day, GD6-delivery) and were subjected to 12-h hypoxia on GD20. Hearts and kidneys were collected from adult female offspring for molecular analyses. CTRL – control, GD – gestational day, PD – postnatal day, PH – prenatal hypoxia, PW – postnatal week, R – restraint. Created with BioRender.com

The first experiment was designed to assess PH effects on cardiovascular function in adult female offspring using *in vivo* telemetry. Pregnant Wistar rats were randomly assigned to normoxic or hypoxic groups. On gestational days (GD) 19 and 20 (sensitive stage of brain development [22]), the hypoxic group (n=6) was exposed to hypoxia (10.5 % O_2_) for 4 h per day. At the same time, normoxic control dams (n=6) were transferred to new cages to ensure similar handling and environmental conditions. Four-month-old female offspring (n=6 per group; one pup per litter) were implanted with telemetry devices under 2–4 % isoflurane anesthesia, and after recovery, their BP and HR were continuously recorded (Fig. 1).

The second experiment focused on the evaluation of molecular changes in offspring’s cardiac and renal tissues and the potential protective effects of maternal ERG treatment. Pregnant Wistar rats were randomly divided into four groups: control (n=6), control+ERG (n=6), hypoxia (n=6), and hypoxia+ERG (n=6). ERG (35 mg/kg/day; dose selected based on previous studies [23,24]) was dissolved in water and administered *via* oral gavage daily from GD6 until delivery. On GD20, pregnant rats were exposed to 12-h hypoxia (10.5 % O_2_). Female offspring (4-month-old) were euthanized at the beginning of the light phase using a lethal dose of anesthesia. Cardiac left ventricles and kidneys were collected, snap-frozen and stored at −80 °C (Fig. 1).

### Western blot

The left ventricles and kidneys were homogenized in a buffer containing sucrose, sodium orthovanadate, sodium fluoride, and protease inhibitor. Total protein concentration was determined by bicinchoninic acid assay (B9643, Sigma-Aldrich, USA). The proteins were denatured at 95 °C and separated on 12 % polyacrylamide gels (120 V, 35 mA, 100 min; 60–75 μg of protein per well). After electrophoresis, proteins were transferred to nitrocellulose membranes (120 V, 200 mA, 60 min; 1620115, BIO-RAD, Germany). Membranes were washed in 5 % bovine serum albumin and incubated overnight at 4 °C with one of the following primary antibodies: anti-beta 1 adrenergic receptor (β_1_-AR; 1:1000, rabbit polyclonal antibody, ab3442, ABCAM; RRID:AB_10890808), anti-cluster of differentiation 68 (CD68; 1:1000, mouse monoclonal antibody, ab31630, ABCAM; RRID:AB_1141557), anti-epithelial sodium channel α (ENaC; 1:1000, rabbit polyclonal antibody, ab214192, ABCAM; RRID:AB_3676284), anti-endothelial nitric oxide synthase (eNOS; 1:1000, mouse monoclonal antibody, ab76198, ABCAM; RRID:AB_1310183), anti-glyceraldehyde-3-phosphate dehydrogenase (GAPDH; 1:5000, mouse monoclonal antibody, MAB374, Millipore; RRID:AB_2107445), anti-myosin light chain kinase (MLCK; 1:1000, rabbit monoclonal antibody, ab76092, ABCAM; RRID:AB_1524000), anti-nuclear factor erythroid 2-rela-ted factor 2 (NRF2; 1:1000, rabbit polyclonal antibody, ab137550, ABCAM; RRID:AB_2687540), anti-sarco/endoplasmic reticulum Ca^2+^-ATPase type 2 (SERCA; 1:1000, rabbit monoclonal antibody, ab150435, ABCAM; RRID:AB_2910256), anti-transforming growth factor beta type 1 (TGF-β1; 1:1000, rabbit monoclonal antibody, ab215715, ABCAM; RRID:AB_2893156). As a secondary antibody, horseradish peroxidase-linked goat anti-rabbit IgG (1:1000; 7074, Cell Signaling; RRID:AB_2099233) or horse anti-mouse IgG (1:5000, 7076, Cell Signaling; RRID:AB_330924) antibodies were used. Protein bands were visualized using ECL (Clarity^TM^ Western ECL Substrate, 170-5061, BIO-RAD, USA) by chemiluminescence (Vü-C Chemiluminescence system, Pop-Bio Imaging, Cambridge, UK) and quantified in Quantity One Basic software (4.6.6., Bio-Rad, USA). The relative intensity of protein bands was normalized to GAPDH expression. Before reprobing, antibodies were removed with stripping buffer (Restore^TM^ Western Blot Stripping Buffer, 21059, Thermo Scientific, Rockford, Illinois, USA).

### Telemetry

Telemetric devices (PA-C40; Data Sciences International, MN, USA) were implanted into the abdominal aorta of 16-week-old female offspring under isoflurane anesthesia (induction: 3–4 % at 1 l/min; maintenance: 1–2 % at 1 l/min). After surgery, animals were subcutaneously administered ampicillin (50 mg/k; BB Pharma, Inc., Prague, Czech Republic) and tramadol (15 mg/kg; Tramal, Stada, Bad Vilbel, Germany). After 2-week recovery period, cardiovascular variables were continuously recorded in 5-min segments every 15 min. Data were sampled at 500 Hz using proprietary software Dataquest A.R.T. version 4.36 gold (DSI, MN, USA).

### Restraint protocol

To assess cardiovascular response to stress, 5-month-old female offspring (n=6 per group) were restrained in transparent plastic tubes (size adjusted to the body size of each rat) for 60 min during the first half of the light phase over three consecutive days. Before restraint, HR and BP were measured for 60 min, and an average of these measurements served as the baseline, to which cardiovascular stress response was normalized. During restraint, animals remained in their home cages, where telemetric recordings were continuously acquired.

### Data evaluation and statistics

From the original continuous 5-min pressure recordings, beat-to-beat intervals (ms) and systolic peaks (mmHg) were extracted and exported as column-separated values files. The time- and frequency-domain parameters of beat-to-beat HR variability (HRV) were assessed using a Lomb-Scargle periodogram within the HRV Analysis Software [25]. Time-domain parameters included the standard deviation of normal-to-normal interbeat intervals (SDNN) and the root mean square of successive differences between interbeat interval differences (RMSSD). Frequency-domain parameters included low-frequency (LF, 0.2–0.75 Hz) and high-frequency (HF, 0.75–2.5 Hz) components [26]. Spontaneous baroreflex sensitivity was assessed as α-index within the LF range ((LF_HRV_/LF_BPV_)^0.5^) [10].

Additional waveform-derived variables were calculated from arterial pressure using the Blood Pressure Analysis Module (DSI, USA). These included time to peak (time from the rise of the systolic pressure to the peak systolic pressure, in ms), ejection time (time from the rise of the systolic pressure to the point of maximum negative pressure derivative), maximum positive pressure derivative (+dP/dt), maximum negative pressure derivative (−dP/dt), and %Recovery (time required for pressure to return from peak to 70 % of diastolic BP).

For the restraint data analysis, the area under the curve (AUC) was computed from data recorded during the whole 60-min restraint protocol according to the formula:


$$\text{AUC}=\sum_{\text{i}=1}^{\text{n}-1}\left(\frac{{\text{Value}}_{\text{i}+1}+{\text{Value}}_{\text{i}}}{2}\times ({\text{Time}}_{\text{i}+1}-{\text{Time}}_{\text{i}})\right)$$

Early (5 min) and late (30 min) ΔHR and ΔBP responses to restraint stress are presented as mean ± SD.

A 24-h variability of HR and BP was analyzed using the Chronos-Fit software [27–29]. The following parameters were evaluated to assess 24-h variability: percentage of rhythm (coefficient of determination ×100; represents the percentage of variation in the data that can be explained by the 24-h model used), mesor (midline estimating statistic of rhythm), amplitude (amplitude of the sinewave), and acrophase (time when the monitored parameter reaches its highest value). Telemetry data are presented as 5-min, 1-h and 12-h mean ± standard deviation (SD).

Statistical significance was determined by unpaired *t*-test (restraint AUC, control vs. PH), two-way ANOVA and two-way ANOVA with repeated measures (factors: hypoxia and time) followed by Bonferroni’s or Fisher’s LSD multiple comparisons tests. Statistical analysis and data visualization were performed using GraphPad Prism version 10.2.3 for macOS (GraphPad Software, Boston, Massachusetts, USA).

## Ethics approval and consent to participate

The experiments were approved by the Ethical Committee for the Care and Use of Laboratory Animals, and the State Veterinary and Food Administration of Slovak Republic (1043-3/2020-220, 4451/3/2022-220), following the EU directive (2010/63/EU) and ARRIVE guidelines.

## Results

### Basal cardiovascular variables

**Fig. 2:**
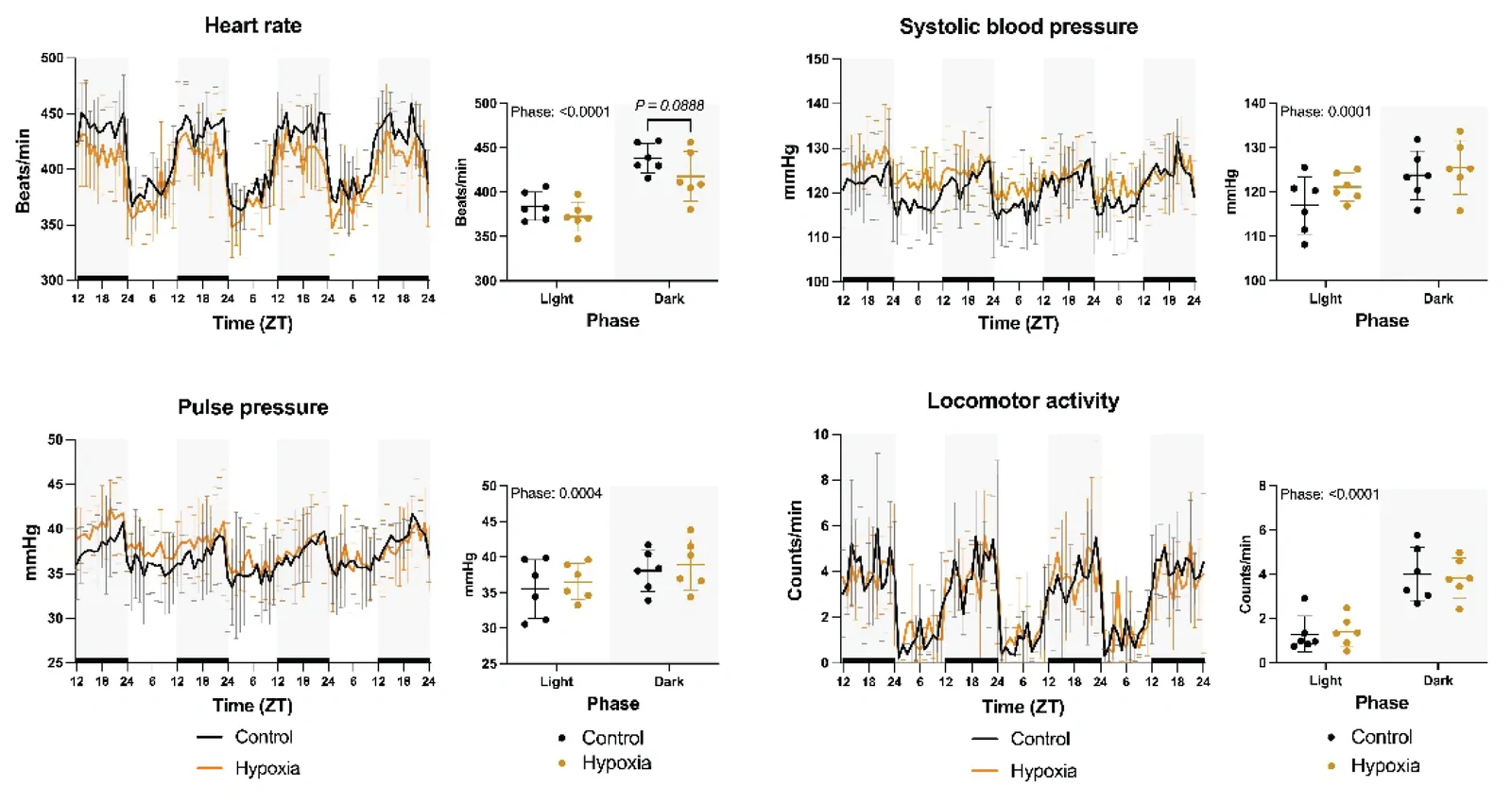
Basal cardiovascular variables and locomotor activity in control and PH female offspring. HR, systolic and diastolic BP, pulse pressure, and locomotor activity were continuously assessed by telemetry. All variables exhibited significant day-night variability. Data are presented as 1-h and 12-h means ± SD. ZT – zeitgeber time. * P<0.05, ** P<0.01, *** P<0.001; Bonferroni’s *post hoc*.

PH had no significant effect on basal HR, systolic BP, diastolic BP, pulse pressure (Table 1), or locomotor activity (control light: 1.3±0.8 counts/min, PH light: 1.4±0.7 counts/min, P>0.999; control dark: 4.0±1.2 counts/min, 3.8±0.9 counts/min, P>0.999; Fig. 2).

The 24-h variability (acrophase, amplitude, mesor and percentage of rhythm) of HR, BP, pulse pressure, and locomotor activity were not affected by PH (Table 2).

In the light phase, PH significantly reduced the absolute HF (P=0.004, Fisher’s LSD) and showed a trend toward reducing the normalized HF (P=0.072, Fisher’s LSD; Table 1). At the same time, normalized LF was increased by PH (P=0.072, Fisher’s LSD), resulting in the increased LF/HF ratio (P=0.051, Fisher’s LSD; Table 1). In the dark phase, PH reduced absolute HF (P=0.011) and showed a trend toward reducing absolute LF (P=0.075), while normalized HF, LF, and LF/HF ratio remained unaffected. Additionally, PH increased SDNN during the dark phase (P=0.024, Fisher’s LSD; Table 1).

PH had no significant effect on the time to peak, ejection time, −dP/dt, +dP/dt, and the %Recovery (Table 3). The spontaneous baroreflex sensitivity exhibited a significant day-night variability in both control and PH groups (P<0.0001) and was not affected by PH (control light: 1.167±0.192 ms/mmHg, PH light: 1.171±0.163 ms/mmHg, P=0.958; control dark: 0.847±0.110 ms/mmHg, PH dark: 0.899±0.114 ms/mmHg; P=0.549).

### Repeated restraint stress

**Fig. 3:**
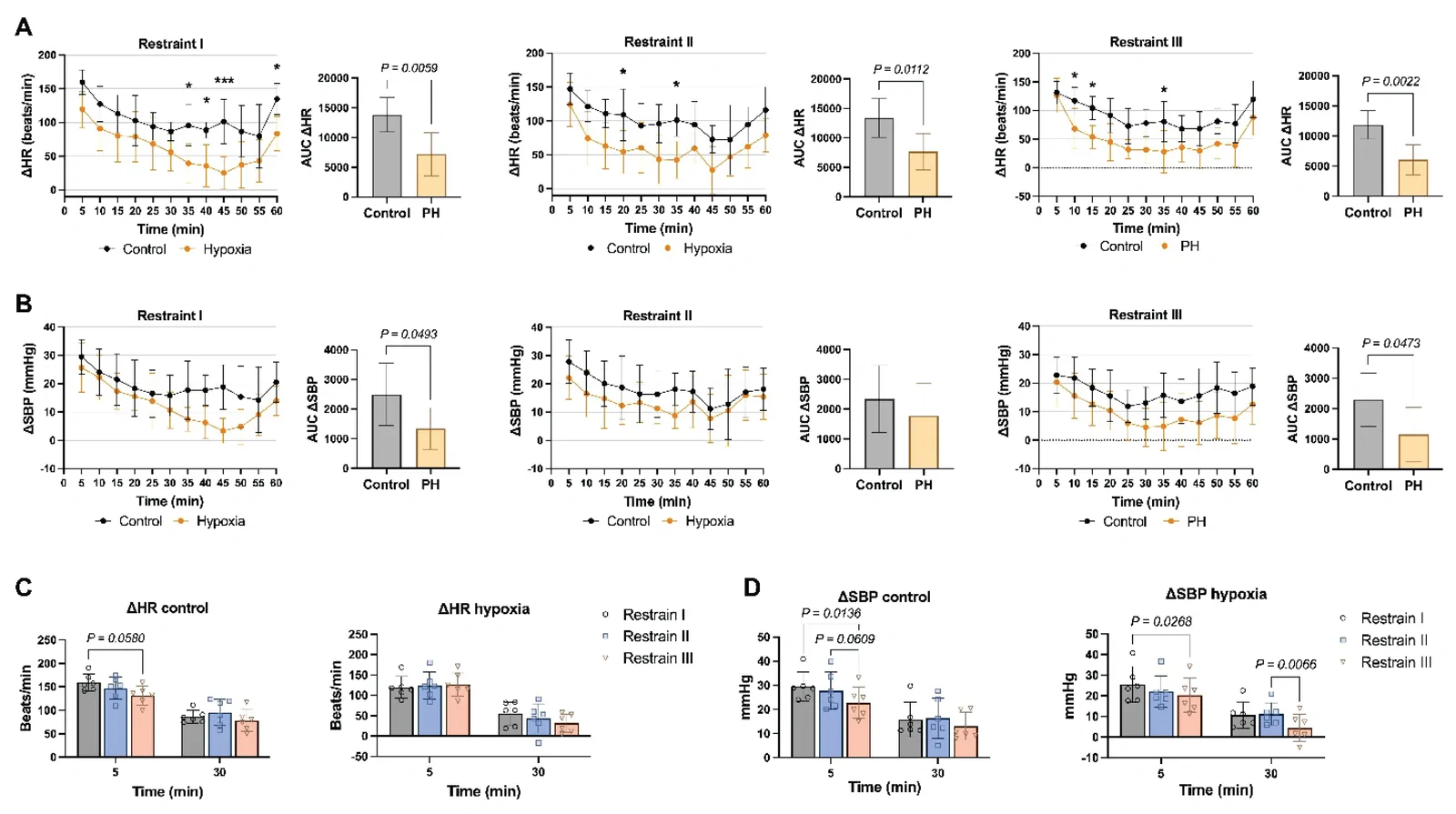
Effects of PH on the cardiovascular response to repeated restraint stress. Adult female offspring were subjected to 60-min restraint for three consecutive days. (**A**) Changes in HR in control and PH offspring during the three restraint sessions. Data are presented as 5-min means ± SD and AUC. (**B**) Changes in systolic BP in control and PH offspring during the three restraint sessions. Data are presented as 5-min means ± SD and AUC. (**C**) Comparison of HR changes between individual restraint sessions at min 5 and 30 in control and PH offspring. Data are presented as individual datapoints and means ± SD. (**D**) Comparison of systolic BP changes between individual restraint sessions at min 5 and 30 in control and PH offspring. Data are presented as individual datapoints and means ± SD. AUC – area under the curve, HR – heart rate, SBP – systolic blood pressure.

During 60-min restraints, HR was significantly lower in PH than in control group (ΔHR AUC day 1 control: 13847±2918, PH: 7230±3630, P=0.006; day 2 control: 13429±3332, PH: 7688±3070, P=0.011; day 3 control: 11827±2359, PH: 6046±2552, P=0.002; two-tailed *t*-test (Fig. 3A). In control group, initial HR response to restraint stress decreased with repeated exposure (ΔHR restraint day 1: 160±18 beats/min, restraint 2: 147±23, restraint day 3: 132±20 beats/min, P=0.017, repeated measures one-way ANOVA). In contrast, PH offspring did not exhibit significant differences in HR response to repeated restraint between the three restraint protocols (ΔHR restraint day 1: 120±27 beats/min, restraint day 2: 124±33 beats/min, restraint day 3: 127±29 beats/min, P=0.650, repeated measures one-way ANOVA; Fig. 3C). After 30 min of restraint, ΔHR was similar across the restraint sessions (ΔHR control: restraint day 1: 87±14 beats/min, restraint day 2: 96±28, restraint day 3: 78±23 beats/min, P=0.440; PH: restraint day 1: 56±28 beats/min, restraint day 2: 44±36, restraint day 3: 32±22 beats/min, P=0.207, repeated measures one-way ANOVA; Fig. 3C).

BP response to restraints was similarly attenuated in PH compared to control group (ΔBP AUC day 1 control: 2497±1053, PH: 1344±698, P=0.049; day 2 control: 2338±1121, PH: 1774±1085, P=0.396; day 3 control: 2298±876, PH: 1150±884, P=0.047, two-tailed *t*-test; Fig. 3B). The initial systolic BP response to stress progressively declined with repeated restraint in both groups (ΔBP control restraint 1: 30±6 mmHg, restraint day 2: 28±8 mmHg, restraint day 3: 23±6 mmHg, P=0.006; PH restraint day 1: 26±9 mmHg, restraint day 2: 22±8 mmHg, restraint day 3: 20±8 mmHg, P=0.017, repeated measures one-way ANOVA; Fig. 3D). After 30 min of restraint, BP responses were similar within the group across all restraints (ΔBP control restraint day 1: 16±7 mmHg, restraint day 2: 16±8 mmHg, restraint day 3: 13±6 mmHg, P=0.453; PH restraint day 1: 11±6 mmHg, restraint day 2: 11±5 mmHg, restraint day 3: 5±7 mmHg, P=0.067, repeated measures one-way ANOVA; Fig. 3D).

### PH effects on cardiac and renal protein expression

**Fig. 4:**
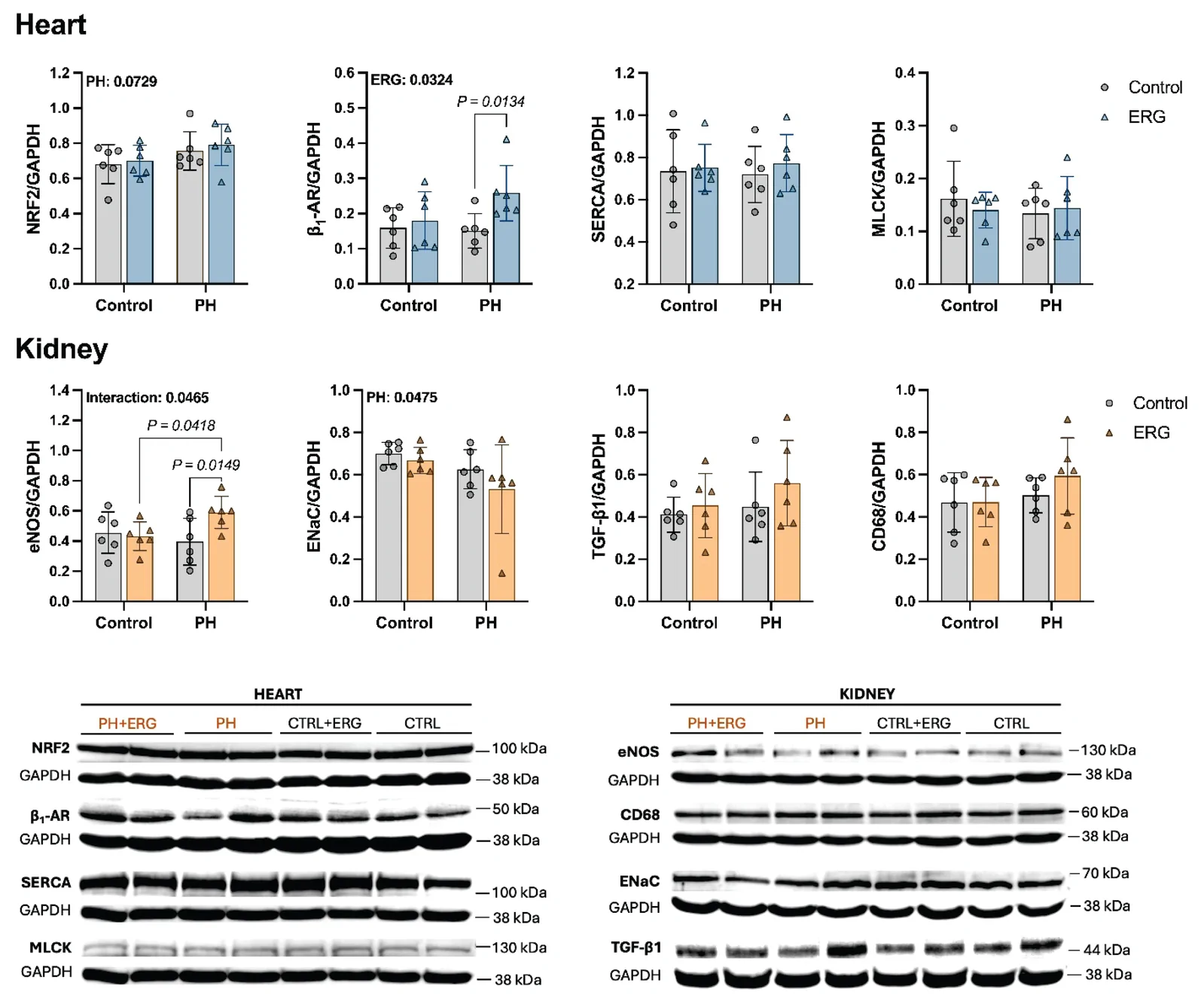
Effects of PH and maternal ERG treatment on cardiac (blue) and renal (yellow) protein expression. Cardiac proteins analyzed included NRF2 (cellular defense against oxidative stress), β_1_-AR (positive chronotropy and inotropy), SERCA, and MLCK (calcium signaling). Renal proteins included eNOS (tubular transport), ENaC (sodium reabsorption), TGF-β1 (fibrosis), and CD68 (macrophage infiltration). Relative protein intensities were normalized to GAPDH. Data are presented as individual datapoints and means ± SD. P values are from Fisher’s LSD *post hoc* test. β_1_-AR – β_1_-adrenergic receptor, CD68 – cluster of differentiation 68, ENaC – epithelial sodium channel α, eNOS – endothelial nitric oxide synthase, ERG – ergothioneine, GAPDH – glyceraldehyde-3-phosphate dehydrogenase, MLCK – myosin light chain kinase, NRF2 – nuclear factor erythroid 2-related factor 2, PH – prenatal hypoxia, SERCA – sarco/endoplasmic reticulum Ca^2+^-ATPase type 2, TGF-β1 – transforming growth factor beta type 1.

In the left ventricle, PH showed a trend towards upregulating NRF2 (P=0.073, two-way ANOVA; Fig. 4). Neither β_1_-AR (P=0.224, two-way ANOVA), MLCK (P=0.597, two-way ANOVA) nor SERCA (P=0.958, two-way ANOVA) expressions were affected by PH.

In the kidneys, PH downregulated ENaC (P=0.048, two-way ANOVA). However, *post hoc* comparisons between control and PH groups did not confirm statistically significant difference (P=0.301, Fisher’s LSD). No significant effects of PH were observed for eNOS (P=0.352, two-way ANOVA), TGF-β1 (P=0.278, two-way ANOVA), or CD68 (P=0.169, two-way ANOVA; Fig. 4).

### ERG effects on cardiac and renal protein expression

In the heart, ERG selectively upregulated β_1_-AR only in PH group (PH vs. PH ERG: P=0.013, Fisher’s LSD; Fig. 4). ERG had no effect on cardiac SERCA (P=0.571, two-way ANOVA), NRF2 (P=0.533, two-way ANOVA) and MLCK (P=0.798, two-way ANOVA) expressions (Fig. 4).

In the kidney, a significant (P=0.047) interaction between PH and ERG was observed for eNOS expression (Fig. 4). *Post hoc* analysis revealed that ERG significantly upregulated eNOS in PH offspring (PH vs. PH ERG: P=0.015, Fisher’s LSD). eNOS expression in PH ERG group was higher than in control ERG offspring (control ERG vs. PH ERG: P=0.042, Fisher’s LSD). ERG had no significant effect on the expression of renal ENaC (P=0.219, two-way ANOVA), TGF-β1 (P=0.237, two-way ANOVA) and CD68 (P=0.409, two-way ANOVA; Fig. 4).

## Discussion

Chronic PH experienced during the embryonic and fetal period increases long-term cardiovascular risk. Our previous studies [10,11] demonstrated that even short episodes of hypoxia during late gestation elevated systolic BP and pulse pressure in male rat offspring. In contrast to males, our present study showed that using the same experimental design (4-h hypoxia on GD19 and GD20, 4-month-old offspring), basal BP remained unaffected by PH in female offspring. Similar sex-specific differences have been reported in the guinea pigs and rats, where late-gestational or chronic intermittent gestational hypoxia increased mean arterial pressure in male, but not female, offspring [19,20]. In contrast to acute PH, chronic PH involving early developmental stages has been shown to increase BP even in female offspring [9,12].

While numerous studies have shown that PH elevates BP, HR appears to remain unaffected [9,11,19,30]. However, in the present study, PH female offspring tended to have a lower HR during the dark (active) phase than did the control group. Furthermore, this subtle reduction became more pronounced during restraint stress, with PH offspring displaying significantly lower HR and systolic BP than controls. These findings are important in the context of previous data from a chronic PH model, which showed an exaggerated HR and BP response to stress [9,31]. The discrepancy may reflect differences in the timing and duration of hypoxic exposure: while chronic PH interferes with organogenesis, acute late-gestational hypoxia may primarily affect maturation of the autonomic nervous system [32] or endocrine signaling [33]. This may explain why acute late-gestational hypoxia yields more subtle and distinct effects than do chronic models.

Given that PH-induced BP changes in male offspring have been linked to increased sympathovagal balance [10], we analyzed whether the cardiovascular profile observed in PH females reflects an autonomic adaptation to PH. Unexpectedly, we observed an increase in the LF component and the LF/HF ratio, along with a reduction in the HF component of HRV during the light phase. These shifts in frequency-domain parameters suggest a trend toward sympathetic dominance during the resting phase, mirroring the pattern previously reported in male offspring prenatally exposed to hypoxia [10]. By contrast, during the dark phase, PH offspring exhibited an increase in total HRV (SDNN). However, no changes were detected in normalized frequency-domain HRV parameters, time to peak, dP/dt, ejection time, %Recovery, or baroreflex sensitivity, suggesting that neither cardiac contractility, vascular compliance, nor baroreflex function are affected by PH in female offspring. Taken together, these findings indicate that PH induces only subtle changes in autonomic regulation in female offspring, characterized by a reduction in vagal tone and an increase in sympathovagal balance during the resting phase. Although these changes resemble those reported in male offspring, the cardiovascular phenotype of PH females differs substantially. Therefore, altered autonomic balance alone cannot account for the observed suppression of HR and BP in PH females, and additional mechanisms must be involved.

One possible mechanism contributing to the lower HR observed in stressed female PH offspring could involve alterations in the cardiac β-adrenergic system or calcium handling. Previous findings from various animal models have shown that prolonged PH upregulates cardiac β_1_-AR, augments the cardiac response to β_1_-AR stimulation, and alters calcium signaling in coronary arteries [34–37]. By contrast, our short-term PH model did not induce any significant changes in cardiac β_1_-AR, SERCA, or MLCK levels in female offspring, suggesting that either the short episode of PH was insufficient to disrupt these regulatory pathways or that females are inherently less susceptible to such alterations. Interestingly, PH upregulated NRF2, a key transcription factor that binds to the antioxidant response element to induce the expression of numerous antioxidant enzymes, while also modulating inflammation and tissue remodelling (reviewed in [38]). Notably, our prior RNA analysis indicated a significant increase in *Nfe2l2* expression in female hearts following 12 h of PH (10.5 % O_2_ on GD20; P=0.034, *t*-test, control vs. PH; unpublished data). A similar upregulation of cardiac *Nfe2l2* has been reported in adult male offspring by Spiroski *et al*. [39] following chronic PH. Furthermore, studies in rodents have shown that NRF2 expression is significantly linked to BP regulation [40,41]. For example, in mice with chronic heart failure, NRF2 upregulation in the rostral ventrolateral medulla reduced sympathetic nerve activity and dampened the BP response to restraint stress [40]. In the context of PH, it is particularly notable that even a short hypoxic exposure limited to the end of gestation can induce redox imbalance and initiate compensatory programming of antioxidant defense pathways. Although the functional significance of NRF2 upregulation in this context remains to be determined, it may reflect a persistent adaptive response aimed at enhancing cardiac resilience to future stress.

Through hypoxia-inducible factor (HIF) signaling, hypoxia also interacts with the circadian system [42]. In a rat model of chronic gestational hypoxia, adult offspring exhibited a phase advance in activity rhythms, delayed resynchronization to a phase delay, and prolonged corticosterone secretion following restraint stress [6]. In our study, short-term PH did not alter circadian variability of HR, BP, or locomotor activity under a regular light/dark cycle, which is consistent with our previous findings in male offspring [10]. While chronic PH can induce lasting disruptions in circadian rhythms, short-term PH may be insufficient to disturb the circadian control of cardiovascular function.

In addition to autonomic and humoral BP regulation, the kidneys play a key role in long-term BP control, primarily through the regulation of sodium balance and extracellular fluid volume. Sodium reabsorption is an energy-demanding process that requires a continuous oxygen supply. Under hypoxic conditions, limited oxygen availability leads to reduced ATP levels and, consequently, attenuated sodium transport. In the present study, we showed that PH downregulated the epithelial sodium channel (ENaC). ENaC is expressed predominantly in the collecting duct, where it mediates the final adjustment of sodium reabsorption. Its expression and activity are increased by aldosterone and vasopressin during hypovolaemia and sodium depletion (reviewed in [43]). Previous studies, both *in vitro* and *in vivo*, have shown that HIF-1α can suppress ENaC expression and activity [44,45]. Whether the slight downregulation observed in female PH offspring could translate into altered sodium handling or BP regulation under dietary or environmental challenges remains to be explored. Other protein markers in the kidneys, including TGF-β1 (a driver of fibrosis), CD68 (a marker of macrophage infiltration), and eNOS (involved in tubular transport regulation), were not affected by PH in the present study. This suggests that short-term PH does not impair renal development and function to the extent typically observed following chronic PH [9,46].

### ERG treatment

The consequences of PH are largely attributed to oxidative stress experienced *in utero* [8,12,37,47]. Given the central role of oxidative stress, several studies have investigated the potential of antioxidant treatment during pregnancy to prevent or reduce PH-induced damage [8,12]. For instance, in an ovine model of late-gestational hypoxia, high doses of vitamin C prevented intrauterine growth restriction and later hypertension in adult offspring [12]. However, the antioxidant doses used in animal studies are typically much higher than what would be considered safe for use during human pregnancy [13,14], thus limiting the direct translatability of these findings. Indeed, clinical trials using lower doses of vitamins C and E in preeclamptic women failed to demonstrate therapeutic benefit [14].

ERG, on the other hand, has emerged as a safe [15] and promising candidate for therapeutic intervention. For instance, in a rat model of preeclampsia, ERG (25 mg/kg) ameliorated maternal hypertension, increased placental NRF2 expression and antioxidant capacity, and improved fetal growth, as indicated by increased pup weights [23] However, its long-term effects on the offspring’s cardiovascular system have not yet been characterized. In our study, ERG was administered to pregnant dams from the early stages of fetal organogenesis (GD6) until delivery, at a dose of 35 mg/kg, resulting in sustained fetal exposure throughout the prenatal period. We observed a strong interaction between treatment outcomes and the prenatal oxygen environment (normoxia vs. hypoxia). Notably, ERG upregulated cardiac β_1_-AR in both control and PH groups, with the increase more pronounced in the PH group. In this context, it is worth noting that Giussani *et al*. [37] demonstrated that chronic PH enhances cardiac responsiveness to isoprenaline (a β_1_-adrenergic agonist) in adult male offspring – an effect that was prevented by maternal vitamin C treatment during pregnancy. Given that β_1_-AR expression is modulated by hormonal signals, it is possible that ERG may act through endocrine pathways. However, no studies to date have examined the effects of ERG on glucocorticoid or thyroid hormone levels, nor have any investigated β_1_-AR expression in adult offspring following maternal antioxidant treatment in the context of acute PH. Therefore, it remains challenging to determine whether this upregulation reflects a compensatory response (e.g., to reduced sympathetic activity or circulating catecholamines), or whether it may contribute to enhanced cardiac responsiveness to stress.

In the kidney, ERG upregulated eNOS exclusively in the PH group. This upregulation suggests that ERG may enhance nitric oxide production, thereby decreasing sodium reabsorption and helping to preserve renal function in PH offspring (reviewed in [48]). Overall, our findings indicate that the long-term effects of prenatal ERG exposure are strongly influenced by the prenatal oxygen environment, with pronounced effects observed in PH offspring and minimal effects in controls. The observed upregulation of cardiac β_1_-AR and renal eNOS in PH offspring may reflect adaptive mechanisms aimed at preserving cardiovascular function and fluid homeostasis under compromised developmental conditions. However, further studies are needed to fully elucidate ERG’s mechanisms of action and long-term efficacy.

This study has several limitations. First, plasma catecholamine, angiotensin II, and aldosterone levels were not measured, limiting our insight into endocrine adaptations to PH and ERG in female offspring. Another important limitation is that cardiovascular variables were not evaluated by telemetry in the ERG group, precluding assessment of the functional impact of ERG treatment on BP or HR regulation. In addition, we did not directly compare the effects of ERG between female and male offspring, and it remains unclear whether similar effects could be achieved with alternative antioxidants administered at equivalent doses.

## Conclusions

Our results indicate that short-term hypoxia at the end of gestation induces subtle yet long-lasting shifts in autonomic regulation, cardiac antioxidant capacity, and renal sodium handling in adult female offspring. Although no HR or BP differences between control and PH groups were observed under basal conditions, exposure to stress revealed a markedly attenuated cardiovascular response in PH offspring. Maternal treatment with ERG increased β_1_-AR and eNOS expression in PH offspring while exerting minimal effects on the normoxic group. To better understand the long-term impact of maternal ERG treatment, future studies should assess cardiovascular reactivity under stress and conduct functional evaluations of cardiac and renal physiology in PH offspring.

## Figures and Tables

**Table 1:** Effect of PH on the HR, BP and frequency- and time-domain HRV.

	Control Light	Dark	P value	PH Light	Dark	P value
*HR (bpm)*	384±16	438±16	<0.001	372±16	418±28#	<0.001
*SBP (mmHg)*	117±6	124±5	<0.001	121±3	126±6	0.007
*DBP (mmHg)*	81±4	86±4	<0.001	85±3	87±3	0.023
*PP (mmHg)*	36±4	38±3	0.004	37±3	39±4	0.006
*LF (ms* *^2^* *)*	0.032±0.006	0.034±0.008	0.299	0.029±0.008	0.027±0.005#	0.214
*nLF*	0.467±0.049	0.521±0.038	0.247	0.553±0.116#	0.570±0.087	0.724
*HF (ms* *^2^* *)*	0.038±0.004	0.032±0.006	0.032	**0.025±0.010** **	**0.020±0.006** *	0.099
*nHF*	0.533±0.049	0.479±0.038	0.247	0.447±0.116#	0.430±0.087	0.724
*LF/HF*	0.955±0.168	1.178±0.184	0.070	1.501±0.719#	1.507±0.505	0.959
*SDNN (ms)*	9.72±1.13	8.39±0.82	0.012	10.25±0.57	**9.55±0.70** *	0.164
*RMSSD (ms)*	5.25±0.68	4.05±0.52	0.005	4.64±0.83	3.97±0.58	0.093

Analyzed by Fisher’s LSD.

# P<0.1 vs. control;

* P<0.05 vs. control;

** P<0.01 vs. control.

DBP – diastolic blood pressure, HF – absolute high-frequency component, HR – heart rate, LF – absolute low-frequency component, LF/HF – low-to-high frequency ratio, nHF – normalized high-frequency component, nLF – normalized low-frequency component, PH – prenatal hypoxia, PP – pulse pressure, RMSSD – root mean square of successive differences between interbeat interval differences, SBP – systolic blood pressure, SDNN – standard deviation of normal-to-normal interbeat intervals.

**Table 2:** Cosinor analysis of HR, systolic BP, pulse pressure and locomotor activity in control and PH offspring.

	Control		PH Mean	SD	P value
	Mean	SD			
*HR*					
*Acrophase (ZT)*	18.7	2.7	16.7	0.5	*0.131*
*Amplitude (beats/min)*	36.5	13.0	36.2	8.3	*0.965*
*Mesor (beats/min)*	411.0	12.7	395.1	19.9	*0.130*
*Rhythm percentage*	36.4	12.5	28.6	15.5	*0.360*
*Systolic BP*					
*Acrophase (ZT)*	18.8	1.0	19.8	3.6	*0.515*
*Amplitude (mmHg)*	4.5	1.8	3.3	1.6	*0.246*
*Mesor (mmHg)*	120.4	5.8	123.4	4.6	*0.349*
*Rhythm percentage*	20.1	12.2	14.5	10.9	*0.426*
*Pulse pressure*					
*Acrophase (ZT)*	20.2	2.1	20.6	3.1	*0.805*
*Amplitude (mmHg)*	2.0	0.8	1.9	0.7	*0.889*
*Mesor (mmHg)*	36.8	3.5	37.7	3.0	*0.630*
*Rhythm percentage*	23.5	11.5	21.1	12.0	*0.730*
*Locomotor activity*					
*Acrophase (ZT)*	18.4	0.7	18.4	0.8	*0.971*
*Amplitude (counts/min)*	1.9	0.5	1.7	0.6	*0.533*
*Mesor (counts/min)*	2.7	1.0	2.6	0.7	*0.949*
*Rhythm percentage*	27.0	7.3	21.2	10.7	*0.297*

Analyzed by unpaired *t*-test. BP – blood pressure, HR – heart rate, PH – prenatal hypoxia, ZT – zeitgeber time.

**Table 3:** Effect of PH on time to peak, ejection time, dP/dt and %Recovery.

	Control light	PH light	P value	Control dark	PH dark	P value
*TTPK (ms)*	32±1	32±2	0.661	30±1	30±2	0.567
*ET (ms)*	57±2	57±2	0.875	53±3	53±2	0.764
*+dP/dt (mmHg/s)*	2252±205	2335±573	0.765	2655±184	2730±703	0.786
*−dP/dt (mmHg/s)*	869±45	901±67	0.527	1025±69	1040±138	0.766
*%Recovery*	76±3	79±4	0.271	65±4	69±6	0.127

Analyzed by Fisher’s LSD. ET – ejection time, PH – prenatal hypoxia, TTPK – time to peak.
